# Predictive Role of the D-Dimer Level in Acute Kidney Injury in Living Donor Liver Transplantation: A Retrospective Observational Cohort Study

**DOI:** 10.3390/jcm11020450

**Published:** 2022-01-16

**Authors:** Jaesik Park, Sung Un Kim, Ho Joong Choi, Sang Hyun Hong, Min Suk Chae

**Affiliations:** 1 Department of Anesthesiology and Pain Medicine, Seoul St. Mary’s Hospital, College of Medicine, The Catholic University of Korea, Seoul 06591, Korea; jaesik.park@gmail.com (J.P.); lifesucks@catholic.ac.kr (S.H.H.); 2 Department of Anesthesiology and Pain Medicine, St. Vincent’s Hospital, College of Medicine, The Catholic University of Korea, Suwon 16247, Korea; ksu6191@daum.net; 3 Department of Surgery, Seoul St. Mary’s Hospital, College of Medicine, The Catholic University of Korea, Seoul 06591, Korea; hopej0126@gmail.com

**Keywords:** D-dimer, acute kidney injury, living donor liver transplantation

## Abstract

This study aimed to determine the association between serum D-dimer levels and the risk of acute kidney injury (AKI) in patients undergoing living donor liver transplantation (LDLT). Clinical data of 675 patients undergoing LDLT were retrospectively analyzed. The exclusion criteria included a history of kidney dysfunction, emergency cases, and missing data. The final study population of 617 patients was divided into the normal and high D-dimer groups (cutoff: 0.5 mg/L). After LDLT, 145 patients (23.5%) developed AKI. A high D-dimer level (>0.5 mg/L) was an independent predictor of postoperative development of AKI in the multivariate analysis when combined with diabetes mellitus [DM], platelet count, and hourly urine output. AKI was significantly higher in the high D-dimer group than in the normal D-dimer group (odds ratio [OR], 2.792; 95% confidence interval [CI], 1.227–6.353). Patients with a high D-dimer exhibited a higher incidence of early allograft dysfunction, longer intensive care unit stay, and a higher mortality rate. These results could improve the risk stratification of postoperative AKI development by encouraging the determination of preoperative D-dimer levels in patients undergoing LDLT.

## 1. Introduction

Living donor liver transplantation (LDLT) is a widely applied surgical treatment for patients with end-stage liver disease (ESLD) [1]. However, the rate of postoperative complications is high; meticulous monitoring and early intervention are important [2]. Acute kidney injury (AKI) is a critical complication after LDLT, with a reported incidence rate of up to 50% [3,4]. Multiple complex factors affect the development of AKI after liver transplantation (LT), including diabetes mellitus (DM), chronic kidney disease (CKD), body mass index (BMI), and Model for End-Stage Liver Disease (MELD) score [3,5,6,7]. Inflammatory biomarkers (i.e., interleukin [IL]-6, IL-10, and C-reactive protein [CRP]) are also associated with the development of AKI [8,9,10]. Because postoperative AKI is independently associated with increased morbidity and mortality [11,12], AKI-related risk factors must be identified before surgery.

D-dimer is a fibrin breakdown product that usually increases in patients with pulmonary embolism (PE), deep vein thrombosis (DVT), or disseminated intravascular coagulation (DIC). A high plasma D-dimer level has been identified as a prognostic factor in critically ill patients. In addition, the D-dimer level increases in patients with liver cirrhosis and is correlated with the severity of liver dysfunction [13,14]. D-dimer is emerging as an early marker of AKI. The D-dimer level significantly increases in patients with AKI and is associated with an increased risk of mortality in pregnant women [15]. In addition, a high D-dimer level has been associated with the development of contrast-induced AKI in patients undergoing percutaneous coronary intervention (PCI) [16]. However, the effects of a high D-dimer level on the development of AKI in patients undergoing LDLT have not been reported.

We investigated the correlation between the D-dimer level and the severity of postoperative AKI, and our results suggest that determining the D-dimer level along with other preoperative factors helps estimate the risk of AKI. In addition, we evaluated the prognostic value of the D-dimer level for postoperative morbidity and mortality in patients undergoing LDLT.

## 2. Patients and Methods

### 2.1. Ethical Considerations

This study was performed in accordance with the ethical standards of the Declaration of Helsinki. Ethical approval was obtained from the Institutional Review Board of Seoul St. Mary’s Hospital (KC20RISI0176; 6 April 2020). A waiver of informed consent was granted for this retrospective study.

### 2.2. Study Population

The medical records were retrospectively collected between January 2009 and February 2020 for 657 patients who underwent LDLT. The exclusion criteria were cadaveric LT, history of kidney dysfunction (i.e., hepatorenal syndrome, CKD, or history of dialysis [17]), emergency cases, age < 19 years, or missing laboratory data. Ultimately, 58 patients were excluded and the remaining 617 patients were included in the analyses.

### 2.3. Living Donor Liver Transplantation

The transplant surgery and general anesthesia were performed by expert surgeons and anesthesiologists, respectively. The surgical techniques and anesthetic management were performed as described previously [18]. Briefly, the piggyback technique was applied, and an inflow modification (i.e., splenic artery ligation, portocaval shunt, or splenectomy) was performed at the surgeon’s discretion. Balanced anesthesia with proper hemodynamic management was supplied. Packed red blood cells (PRBCs) were transfused to maintain hematocrit >25%, and fresh-frozen plasma (FFP) or single-donor platelets (SDPs) were transfused under the guidance of thromboelastography or according to the laboratory results [9]. Immunosuppressants were administered and were tapered after surgery in accordance with our hospital’s protocol.

### 2.4. Acute Kidney Injury

Postoperative AKI was diagnosed based on the Kidney Disease Improving Global Outcomes criteria [19]. AKI severity was determined as follows: Stage 1, increase in serum creatinine (sCr) ≥ 0.3 mg/dL (in 48 h) or 1.5–1.9 multiplied by the baseline (in 7 days); stage 2, sCr 2.0–2.9 multiplied by the baseline; and stage 3, sCr ≥ 3.0 multiplied by the baseline or an increase in sCr ≥ 4.0 mg/dL at the beginning of renal replacement therapy [20]. The study population was divided into non-AKI and AKI groups.

### 2.5. Measurement of Laboratory Data

Laboratory parameters were evaluated preoperatively. Blood samples were collected into a Clot Activator tube (Becton Dickinson and Co., Franklin Lakes, NJ, USA) and analyzed using an automated chemistry analyzer (Hitachi 7600; Hitachi, Tokyo, Japan) to obtain the laboratory data on the day before surgery. A Sodium Citrate Tube (Becton Dickinson and Co.) and automated coagulation analyzer (CS-5100; SysmexCrop., Kobe, Japan) were used to measure D-dimer levels. A high D-dimer level was defined as >0.5 mg/L fibrinogen equivalent units, as described previously [21]. If samples were collected several times, the one collected nearest the time of surgery was analyzed.

### 2.6. Perioperative Recipient and Donor Graft Factors

The preoperative recipient factors included etiology, BMI, comorbidities (i.e., DM and hypertension), sex, age, MELD score, hepatic decompensation (i.e., ascites, varix, and West-Haven criteria on hepatic encephalopathy [22]), echocardiography (i.e., ejection fraction and diastolic dysfunction [23]), estimated glomerular filtration rate, and laboratory variables (i.e., white blood cell [WBC] count, platelet count, and levels of Cr, glucose, ammonia, potassium, calcium, and albumin). Intraoperative recipient factors included the presence of postreperfusion syndrome [24], average vital signs during surgery (i.e., mean blood pressure, central venous pressure, and heart rate), the volume of blood products transfused (i.e., PRBCs, FFP, or SDPs), mean lactate, surgical duration, urine output, and hourly fluid infusion. Donor graft factors included donor graft fatty change (%), the graft recipient weight ratio, sex, and age.

### 2.7. Clinical Postoperative Outcomes

Postoperative outcomes included the duration of hospital stay, the duration of intensive care unit (ICU) stay, the incidence of early allograft dysfunction (EAD), graft rejection, and overall mortality. EAD was clinically determined by the presence of at least one of the following: total bilirubin ≥10  mg/dL or international normalized ratio (INR) ≥ 1.6 on postoperative day 7 and alanine transaminase or aspartate transaminase ≥ 2000 IU/mL during the first week after surgery [25].

### 2.8. Statistical Analysis

The Mann–Whitney U test, χ^2^ test, or Fisher’s exact test were used to compare perioperative recipient and donor graft factors between the AKI and non-AKI groups. Tests for trends were conducted using a linear-by-linear association method. The values were expressed as the median (interquartile range [IQR]) and number (proportion). The associations between perioperative factors and the development of postoperative AKI were analyzed using logistic regression. Potentially significant factors (*p* < 0.1) in the univariate analysis were included in multivariate forward and backward logistic regression analyses. We conducted two separate multivariate analyses using the dichotomous or continuous D-dimer levels. Dichotomous D-dimer was included in the predictive multivariate model. The predictive performance of the models was estimated by the area under the receiver operating characteristic curve analysis. The relationships between the D-dimer level and inflammatory factors or the MELD were detected using Spearman’s rank correlation coefficient analysis. A *p*-value < 0.05 was considered statistically significant. Statistical analyses were performed using MedCalc (ver. 11.0; MedCalc Software, Ostend, Belgium) and SPSS Statistics software (ver. 24.0; SPSS Inc., Chicago, IL, USA).

## 3. Results

### 3.1. Demographic Characteristics of Patients Undergoing LDLT

The study population (*n* = 617) consisted of 432 men (70%) and 185 women (30%). The LDLT etiologies included hepatitis B (HBV) (56.6%), alcoholic hepatitis (19.6%), hepatitis C (7.3%), cryptogenic hepatitis (6.2%), hepatitis A (4.2%), autoimmune hepatitis (4.2%), and drug-induced and toxic hepatitis (1.9%). The prevalence rates of the preoperative variables were hypertension (20.3%), encephalopathy (8.9%), diabetes (26.3%), varix (24.3%), and ascites (47.3%). The median (IQR) age, MELD score, BMI, and D-dimer level were 54 (48–59) years, 13.6 (6.6–23.7) points, 24 (22–27) kg/m^2^, and 3.6 (1.1–7.4) mg/L, respectively. Among the 617 patients, 145 (23.5%) developed AKI after LDLT.

### 3.2. Analysis of Pre- and Intraoperative Clinical Data According to the Development of AKI

Intergroup differences in the preoperative recipient factors (i.e., MELD score, the incidence of ascites, DM, hemoglobin level, albumin level, platelet count, INR, and D-dimer level) were detected (Table 1). Differences in intraoperative recipient factors (i.e., total PRBC, FFP, and platelet transfusion amounts; average HR; average MBP; and hourly urine output) and donor graft factors (i.e., graft ischemic time) were observed between the groups (Table 2).

### 3.3. Associations of Pre- and Intraoperative Factors with the Development of AKI

The D-dimer level was significantly associated with the development of AKI in the multivariate logistic regression (Table 3), when combined with the presence of DM, platelet count, and intraoperative hourly urine output (AUC: 0.672; 95% confidence interval (CI): 0.634–0.709; sensitivity: 72.4%; specificity: 55.9%; *p* < 0.001). Furthermore, the probability that patients with a high D-dimer level (>0.5 mg/L) would develop AKI was 2.79-fold higher than in those with a normal D-dimer level in the multivariate analysis using dichotomous D-dimer (odds ratio: 2.792; 95% CI: 1.227–6.353; *p* = 0.014) (Table S1).

### 3.4. Analysis Using the Alternative D-Dimer Cutoff Level

**Figure 1:**
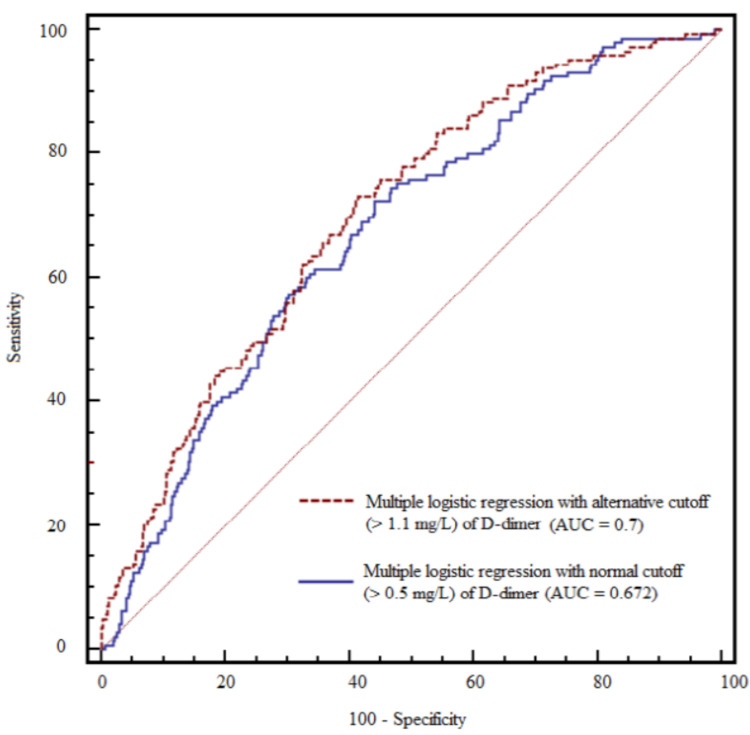
Comparison of the area under the receiver operating characteristic (ROC) curve (AUC) between multiple logistic regression models with the normal (0.5 mg/L) and alternative (1.1 mg/L) D-dimer cutoff levels.

Patients were divided into normal and high D-dimer groups using an alternative cutoff level (>1.1 mg/L) for the development of AKI (AUC: 0.7, 95% CI: 0.662–0.736; sensitivity: 73.1%, specificity: 58.5%, *p* < 0.001) (Figure 1). A high D-dimer level (>1.1 mg/L) was associated with a four-fold increased risk of AKI compared to those with a D-dimer level below the cutoff (odds ratio: 4.025; 95% CI: 2.093–7.744; *p* < 0.001).

### 3.5. Comparison of the Prevalence of AKI Stages between the Normal and High D-Dimer Groups

The prevalence of patients with postoperative AKI was higher in the high D-dimer group than in the normal group for every stage (stage 1 and stages 2–3) (*p* < 0.001; Table 4).

### 3.6. Comparison of D-Dimer Level According to the AKI Stage

**Figure 2:**
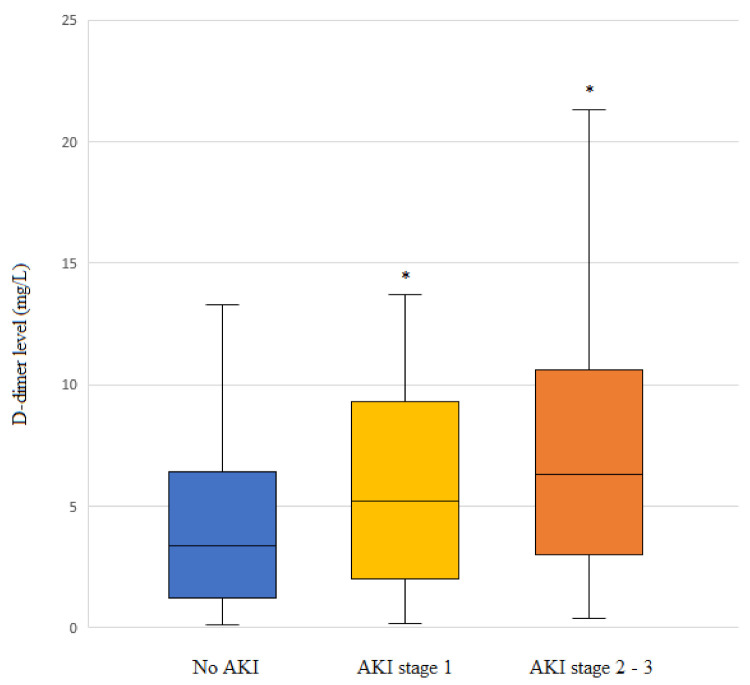
D-dimer levels according to acute kidney injury (AKI) stage in patients undergoing living-donor liver transplantation (LDLT). Box plots show the median (line in the middle of the box), interquartile range (box), and 5th and 95th percentiles (whiskers). * *p* < 0.001 vs. no AKI.

Higher D-dimer levels were detected in patients with higher AKI stages (Figure 2). The median (IQR) D-dimer levels were 3.0 (0.8–6.5), 5.2 (2.0–9.2), and 6.3 (3.0–10.6) mg/L in the non-AKI, AKI stage 1, and AKI stage 2–3 groups, respectively.

### 3.7. Relationships of the D-Dimer Level with Inflammatory Factors

The D-dimer level was significantly correlated with inflammatory factors, including WBC (Rho coefficient = 0.282), albumin (Rho coefficient = −0.356), and CRP (Rho coefficient = 0.575) (all *p* < 0.001).

### 3.8. Relationship between the D-Dimer Level and the MELD Score

The D-dimer level was significantly correlated with the MELD score (Rho coefficient = 0.483) (*p* < 0.001).

### 3.9. Subanalysis of Patients with DM, HBV, or Heart Disease

The prevalence of AKI did not significantly differ between the normal and high D-dimer patients with DM when using the normal (0.5 mg/L) cutoff level. However, a significant difference (*p* = 0.008) was detected when using the alternative (1.1 mg/L) D-dimer cutoff level (Table S2). A significant difference in D-dimer level was observed between the AKI and non-AKI groups of patients with DM (Table S3). There were differences between patients with HBV and heart disease (diastolic dysfunction) with AKI vs. non-AKI and between such patients with a normal D-dimer level vs. a high D-dimer level (Tables S4–S7).

### 3.10. Postoperative Outcomes

Patients with a high D-dimer level had a longer ICU stay, higher incidence of EAD, and a higher mortality rate than did patients with a normal D-dimer level (*p* = 0.036, *p* = 0.005, and *p* = 0.02, respectively) (Table 5). Patients with AKI had a longer duration of hospitalization, longer ICU stay, higher incidence of EAD, and higher overall patient mortality rate than did patients without AKI (*p* < 0.001, *p* = 0.002, *p* = 0.002, and *p* = 0.013, respectively) (Table 6).

## 4. Discussion

The main finding of this study was that a high D-dimer level (>0.5 mg/L) was an independent predictor of postoperative development of AKI when combined with platelet count, DM, and hourly urine output. The prevalence of AKI was significantly higher in the high D-dimer group than in the normal D-dimer group. The D-dimer level and the proportion of patients with a high D-dimer level significantly increased with more severe AKI. In addition, patients with a high D-dimer level or AKI had more severe morbidities and higher mortality rates.

AKI is a common postoperative complication in LT recipients [3]. Although the etiology has not been fully elucidated, renal hypoperfusion, inflammation, hypovolemia, and the use of nephrotoxic drugs are possible contributing factors [26,27]. Recent studies have shown that the systemic inflammatory response plays a critical role in the development of AKI [28,29]. Systemic inflammation causes AKI by targeting tubular epithelial cells (TECs). Inflammatory mediators may contribute to reducing the blood flow in the outer medulla of the kidneys [30]. Serum CRP impairs G1/S-dependent TEC regeneration, while IL-6 and tumor necrosis factor-alpha (TNF-α) interact with TECs, causing renal injury [31,32]. Immune responses also contribute to renal damage after reperfusion injury [33]. The inflammatory reaction of the innate and adaptive immune systems in the post-ischemic kidney is an important factor in the pathogenesis of ischemia-reperfusion injury (IRI). Postoperative AKI is a major risk factor for morbidity and mortality after LDLT and is important for predicting the development of AKI [34]. In the present study, the AKI group had a higher incidence of EAD, longer hospital and ICU stays, and lower overall survival than the non-AKI group.

D-dimer is a fibrin degradation product that is widely measured to aid in the diagnosis of PE. Although D-dimer is a well-known marker for clotting disorders, such as PE, DIC, and DVT, recent studies have shown that a high D-dimer level is also associated with the prognosis of critically ill patients [35,36]. A high D-dimer level is an emerging marker for kidney dysfunction. In patients with ST-elevation myocardial infarction undergoing PCI, D-dimer levels >0.69 μg/mL are an independent predictor of contrast-induced AKI [16]. A retrospective study revealed that the D-dimer level was significantly higher in pregnant women with AKI and was associated with higher mortality risk [15]. In a study of patients undergoing hematopoietic cell transplantation, a high D-dimer level caused by activation of the coagulation system was associated with the development of AKI [37]. Although the mechanism underlying the relationship between D-dimer and AKI is unclear, a high D-dimer level may exacerbate inflammatory coagulation and fibrinolysis (via inflammatory mediator surges, thrombin generation, fibrin formation/degradation, and platelet aggregation), thus contributing to the development of AKI in critically ill patients [38,39,40]. Therefore, the close relationship between the D-dimer level and inflammation may play a role in kidney damage. In the present study, a high D-dimer level was correlated with more severe liver dysfunction [13,14,41], suggesting its use as an early surrogate marker for hepatic decompensation that increases the risk of AKI in patients scheduled for LDLT. 

Our multivariate logistic analysis suggested that other risk factors for postoperative AKI include preoperative DM, platelet count, and intraoperative hourly urine output. A diabetic kidney has decreased resilience to renal perfusion after ischemia. Habib et al. [42] suggested that delayed reperfusion in the renal cortex and increased apoptosis of proximal tubular cells were possible mechanisms of renal IRI in the diabetic kidney. Additionally, microvascular dysfunction may inhibit renal perfusion after ischemia [43]. The preoperative platelet count was a preoperative risk factor for the development of AKI in the present study. In a study of elderly patients, thrombocytopenia was associated with an increased risk of AKI [44]. The probability of AKI significantly increased in patients with severe thrombocytopenia after LDLT [45]. Considering the close relationship between portal hypertension and thrombocytopenia, a low platelet count may be associated with portal hypertension-related renal injury in patients with ESLD. A decrease in the hourly urine output during surgery was also significantly associated with postoperative AKI. Low urine output usually indicates hypovolemia or hypotension, both of which are related to decreased perfusion pressure in the afferent arterioles [46]. Intraoperative oliguria is associated with the development of postoperative AKI in patients undergoing major abdominal surgery [47]. Reduced urine output was associated with an increased risk of AKI in an ICU-based study [48].

Several limitations of this study should be discussed. First, the pathophysiological processes underlying the association between an elevated D-dimer level and the development of AKI are unknown. Although a high D-dimer level is associated with systemic inflammation, further studies are required. Second, we used the D-dimer reference range based on clotting disorders, such as PE. Further studies are required to establish the D-dimer range in the AKI setting. Third, hidden biases could have been present because this study constituted a retrospective analysis. Fourth, liver grafts from deceased donors are more closely associated with postoperative AKI than grafts from living donors [49]. Therefore, the association between the D-dimer level and the development of AKI could differ according to the donor type. Fifth, we could not include the length of waiting time for LT or the liver disease duration. These periods would provide important information because a patient’s condition could become worse with a longer waiting time and longer duration of cirrhosis. However, all donors were relative volunteers (parents, sister, and brother); this aspect may have contributed to the shorter wait time and cirrhotic duration than in LDLT with other types of donations or DDLT [50,51]. The MELD score played a key clinical role in assessing the severity of cirrhosis [52,53].

## 5. Conclusions

The findings in this study suggest that a high D-dimer level (>0.5 mg/L) constitutes an independent predictor of postoperative development of AKI when combined with DM, platelet count, and hourly urine output. Because its odds ratio (2.792) was highest among those risk factors, the D-dimer level could provide critical information regarding vulnerability to AKI. Our findings suggest that a high D-dimer level is an early and promising marker for the development of AKI; it could provide useful information for understanding a patient’s condition. Risk factors for the development of AKI, including the D-dimer level, must be assessed before surgery; patients with those risk factors should be carefully monitored.

## Figures and Tables

**Table 1:** Comparison of preoperative recipient clinical factors between the non-AKI and AKI groups.

Group	Non-AKI	AKI	*p*
*n*	472	145	
**Preoperative recipient factor**			
Age (years)	54 (48–59)	52 (48–58)	0.297
Sex (male)	324 (68.6%)	108 (74.5%)	0.180
Body mass index (kg/m^2^)	24 (22–26)	24 (22–28)	0.118
Etiology			
Alcohol	84 (17.8%)	37 (25.5%)	0.268
Hepatitis A	18 (3.8%)	8 (5.5%)	
Hepatitis B	270 (57.2%)	79 (54.5%)	
Hepatitis C	37 (7.8%)	8 (5.5%)	
Autoimmune	23 (4.9%)	3 (2.1%)	
Drug & Toxin	10 (2.1%)	2 (1.4%)	
Cryptogenic	30 (6.4%)	8 (5.5%)	
Comorbidity			
Diabetes mellitus	114 (24.2%)	48 (33.1%)	0.032
Hypertension	95 (20.1%)	30 (20.7%)	0.883
MELD score (point)	12 (6–23)	17 (10–25)	0.007
Hepatic decompensation			
Encephalopathy (West-Haven criteria I or II)	38 (8.1%)	17 (11.7%)	0.175
Varix	111 (23.5%)	39 (26.9%)	0.407
Ascites	209 (44.3%)	83 (57.2%)	0.006
Cardiac function			
Ejection fraction (%)	64 (62–67)	64 (62–67)	0.303
Diastolic dysfunction	193 (40.9%)	65 (44.8%)	0.4
Renal function			
eGFR (mL/min/1.73 m^2^)	90.5 (77.0–114.1)	87.4 (74.1–117.2)	0.832
Laboratory variables			
Hemoglobin (g/dL)	10 (8.4–12)	9.4 (8.1–10.8)	0.008
WBC count (×10^9^/L)	4.3 (2.8–6.8)	4.6 (3.1–8)	0.249
Albumin (g/dL)	3.1 (2.7–3.6)	2.9 (2.6–3.3)	0.001
Platelet count (×10^9^/L)	67.5 (47–110)	55 (39–77)	<0.001
International normalized ratio	1.4 (1.2–2.1)	1.6 (1.3–2.1)	0.025
Activated partial thrombin time (sec)	35.3 (28.5–51)	33.5 (29–46)	0.156
D-dimer (mg/L)	3 (0.8–6.5)	5.4 (2.5–9.8)	<0.001
Sodium (mEq/L)	139 (135–142)	138 (135–141)	0.078
Potassium (mEq/L)	4 (3.7–4.3)	4 (3.6–4.3)	0.362
Calcium (mg/dL)	8.4 (8–8.8)	8.4 (7.9–8.8)	0.307
Glucose (mg/dL)	108 (91–139)	112 (96–142)	0.218
Creatinine (mg/dL)	0.9 (0.7–1.1)	0.9 (0.7–1.3)	0.589
Ammonia (μg/dL)	95 (65–148)	105 (68–160)	0.442

Abbreviations: AKI, acute kidney injury; eGFR, estimated glomerular filtration rate; MELD, Model for End-stage Liver Disease; WBC, white blood cell. NOTE: Values are medians (ranges) or numbers (percentages).

**Table 2:** Comparison of intraoperative recipient and donor clinical factors between the non-AKI and AKI groups.

Group	Non-AKI	AKI	*p*
*n*	472	145	
**Intraoperative recipient factor**			
Surgical duration (min)	505 (450–570)	510 (455–584)	0.435
Postreperfusion syndrome	245 (51.9%)	83 (57.2%)	0.260
Average of vital signs			
MBP (mmHg)	76.3 (70.5–83)	75 (68.4–82)	0.038
HR (beats/min)	88 (80–99)	92 (82–102)	0.035
CVP (mmHg)	9 (7.1–10.8)	9.3 (7.1–11.5)	0.397
Mean lactate (mmol/L)	3.7 (2.8–4.8)	3.7 (2.8–5.5)	0.732
Blood product transfusion (unit)			
Packed red blood cell	6 (3–12)	10 (6–15)	<0.001
Fresh frozen plasma	6 (4–10)	10 (6–13)	<0.001
Platelet concentrate	3 (0–10)	6 (0–12)	0.019
Hourly fluid infusion (mL/kg/h)	10.2 (7.6–13.3)	10.7 (8.1–14.3)	0.253
Hourly urine output (mL/kg/h)	1.5 (0.8–2.3)	1.1 (0.5–1.8)	<0.001
Donor-graft factor			
Age (years)	32 (25–43)	32 (24–48)	0.606
Sex(male)	256 (61%)	69 (63.3%)	0.653
GRWR (%)	1.2 (1–1.4)	1.2 (1–1.4)	0.105
Graft ischemic time (min)	90 (70–117)	101 (79–148)	<0.001
Warm ischemic time (min)	31 (26–42)	35 (27–45)	0.266
Cold ischemic time (min)	50 (36–78)	70 (39–130)	0.012
Fatty change (%)	5 (1–5)	4 (0–5)	0.540

Abbreviations: AKI, acute kidney injury; CVP, central venous pressure; GRWR, graft recipient weight ratio; HR, heart rate; MBP, mean blood pressure. NOTE: Values are medians (interquartile ranges) or numbers (percentages), unless indicated otherwise.

**Table 3:** Associations of pre- and intraoperative factors with the occurrence of AKI in living donor liver transplantation.

	Univariable Analysis				Multivariable Analysis			
	β	Odds Ratio	95% CI	*p*	β	Odds Ratio	95% CI	*p*
**Preoperative recipient factor**								
Age (years)	−0.005	0.995	0.976–1.015	0.648				
Sex (male vs. female)	−0.288	0.750	0.492–1.143	0.180				
Body mass index (kg/m^2^)	0.042	1.042	0.994–1.093	0.085				
Comorbidity								
Diabetes mellitus	0.441	1.554	1.036–2.330	0.033	0.425	1.529	1.005–2.327	0.048
Hypertension	0.035	1.035	0.653–1.641	0.883				
MELD score (point)	0.018	1.018	1.001–1.035	0.035				
Hepatic decompensation								
Encephalopathy (West-Haven criteria I or II)	0.417	1.517	0.828–2.778	0.177				
Varix	0.179	1.197	0.783–1.829	0.407				
Ascites	0.522	1.685	1.157–2.453	0.007				
Aspartate aminotransferase	0.000	1.000	0.999–1.000	0.333				
Alanine aminotransferase	0.000	1.000	1.000–1.000	0.445				
Cardiac function								
Ejection fraction (%)	0.029	1.029	0.987–1.072	0.175				
Diastolic dysfunction	0.161	1.175	0.807–1.709	0.401				
Renal function								
eGFR (mL/min/1.73 m^2^)	0.002	1.002	0.997–1.006	0.510				
Laboratory variables								
Hemoglobin (g/dL)	−0.116	0.891	0.816–0.973	0.010				
WBC count (×10^9^/L)	0.017	1.017	0.986–1.049	0.288				
Albumin (g/dL)	−0.564	0.569	0.408–0.793	0.001				
Platelet count (×10^9^/L)	−0.007	0.993	0.989–0.997	0.001	−0.005	0.995	0.990-0.999	0.013
International normalized ratio	0.126	1.135	0.913–1.410	0.255				
Activated partial thrombin time (sec)	0.004	1.004	0.986–1.022	0.686				
D-dimer (mg/L)	0.048	1.050	1.023–1.077	<0.001	0.032	1.032	1.004–1.061	0.026
Sodium (mEq/L)	−0.020	0.980	0.947–1.014	0.240				
Potassium (mEq/L)	−0.138	0.871	0.636–1.192	0.388				
Calcium (mg/dL)	−0.116	0.891	0.697–1.138	0.355				
Glucose (mg/dL)	0.001	1.001	0.998–1.004	0.505				
Creatinine (mg/dL)	−0.135	0.873	0.723–1.055	0.161				
Ammonia (μg/dL)	0.001	1.001	0.999–1.003	0.338				
**Intraoperative recipient factor**								
Surgical duration (min)	0.001	1.001	0.999–1.003	0.361				
Postreperfusion syndrome	0.215	1.240	0.852–1.805	0.261				
Average of vital signs								
MBP (mmHg)	−0.018	0.982	0.964–1.001	0.060				
HR (beats/min)	0.010	1.010	0.998–1.021	0.096				
CVP (mmHg)	0.043	1.044	0.985–1.107	0.148				
Mean lactate (mmol/L)	0.035	1.036	0.972–1.104	0.280				
Blood product transfusion (unit)								
Packed red blood cell	0.029	1.030	1.009–1.050	0.004				
Fresh frozen plasma	0.031	1.031	1.006–1.057	0.014				
Platelet concentrate	0.001	1.001	0.988–1.013	0.915				
Hourly fluid infusion (mL/kg/h)	0.011	1.011	0.994–1.029	0.198				
Hourly urine output (mL/kg/h)	−0.464	0.629	0.511–0.773	<0.001	−0.373	0.689	0.558–0.851	0.001
**Donor-graft factor**								
Age (years)	−0.003	0.997	0.980–1.014	0.740				
Sex(male)	−0.100	0.905	0.585–1.400	0.653				
GRWR (%)	0.226	1.253	0.857–1.832	0.244				
Graft ischemic time (min)	0.003	1.003	1.001–1.004	<0.001				
Fatty change (%)	0.006	1.006	0.979–1.006	0.669				

Abbreviations: CI, confidence interval; CVP, central venous pressure; eGFR, estimated glomerular filtration rate; GRWR, graft-recipient weight ratio; HR, heart rate; MBP, mean blood pressure; MELD, Model for End-Stage Liver Disease; WBC, white blood cell.

**Table 4:** Comparison of the prevalence of AKI stages between the normal and high D-dimer groups.

Group	Normal D-Dimer (≤0.5 mg/L)	High D-Dimer (>0.5 mg/L)	*p*
*n*	93	524	
Normal kidney function	86 (92.5%)	386 (73.7%)	<0.001
Mild AKI (stage 1)	6 (6.5%)	86 (16.4%)	
Moderate to severe AKI (stage 2–3)	1 (1.1%)	52 (9.9%) *	

Abbreviations: AKI, acute kidney injury. * *p* < 0.05 using linear by linear method. NOTE: Values are expressed as numbers (with % proportion).

**Table 5:** Comparison of postoperative outcomes between the normal and high D-dimer groups.

Group	Normal D-Dimer (≤0.5 mg/L)	High D-Dimer (>0.5 mg/L)	*p*
n	93	524	
ICU stay (day)	7 (5–7)	7 (6–7)	0.036
Hospital stay (day)	26.5 (21–35)	26 (21–38)	0.554
Graft rejection	23 (13.1%)	152 (29%)	0.399
Early allograft dysfunction	5 (5.4%)	87 (16.6%)	0.005
Overall patient mortality	7 (7.5%)	89 (17%)	0.02

Abbreviations: ICU, intensive care unit. Note: Values are expressed as medians (interquartile ranges) or numbers (percentages).

**Table 6:** Comparison of postoperative outcomes between the non-AKI and AKI groups.

Group	No AKI	AKI	*p*
n	472	145	
ICU stay (day)	7 (6–7)	7 (6–8)	0.002
Hospital stay (day)	25 (21–34)	30.5 (22–47)	<0.001
Graft rejection	136 (28.8%)	39 (26.9%)	0.654
Early allograft dysfunction	59 (12.5%)	33 (22.8%)	0.002
Overall patient mortality	64 (13.6%)	32 (23.5%)	0.013

Abbreviations: AKI, acute kidney injury. Note: Values are expressed as medians (with interquartile range) or numbers (with % proportion).

## Data Availability

The data presented in this study are available on reasonable request.

## Supplementary Materials

The following supporting information can be downloaded at: https://www.mdpi.com/article/10.3390/jcm11020450/s1, Table S1. Associations of pre- and intraoperative factors with the occurrence of AKI in living donor liver transplantation. Table S2. Comparison of the prevalences of AKI between the normal and high D-dimer groups of DM patients. Table S3. Comparison of the D-dimer levels between the non-AKI and AKI groups of DM patients. Table S4. Comparison of the prevalences of AKI between the normal and high D-dimer groups of patients with hepatitis B virus. Table S5. Comparison of the D-dimer levels between the non-AKI and AKI groups of patients with hepatitis B virus. Table S6. Comparison of the prevalences of AKI between the normal and high D-dimer groups of patients with heart disease (diastolic dysfunction). Table S7. Comparison of the D-dimer levels between the non-AKI and AKI groups of patients with heart disease (diastolic dysfunction).

## Abbreviations

LDLT	living donor liver transplantation
ESLD	end stage liver disease
AKI	acute kidney injury
EAD	early allograft dysfunction
CKD	chronic kidney dysfunction
CRP	C-reactive protein
MBP	mean blood pressure
CVP	central venous pressure
DM	diabetes mellitus
DVT	deep vein thrombosis
DIC	disseminated intravascular coagulation
PRBC	packed red blood cell
FFP	fresh frozen plasma
SDP	single donor platelet
PCI	percutaneous coronary intervention
POD	postoperative day
PE	pulmonary embolism
MELD score	Model for end-stage liver disease score
BMI	body mass index
CRRT	continuous renal replacement therapy
HR	heart rate
IQR	interquartile
LT	liver transplantation
DDLT	deceased donor liver transplantation
WBC	white blood cells
