# Supplementary material for: Predictive Role of the D-Dimer Level in Acute Kidney Injury in Living Donor Liver Transplantation: A Retrospective Observational Cohort Study
Source: J Clin Med. 2022 Jan 16;11(2):450. doi: 10.3390/jcm11020450 (PMC8779454; doi:10.3390/jcm11020450)
Supplement: Supplementary file 1 [file jcm-11-00450-s001.zip › jcm-1480027-supplementary.pdf]

Table S1. Associations of pre- and intraoperative factors with the occurrence of AKI in living donor liver transplantation.

	Univariable analysis				Multivariable analysis			
	β	**Odds ratio**	**95% CI**	***p***	***β***	**Odds ratio**	**95% CI**	***p***
**Preoperative recipient factor**								
Age (years)	-0.005	0.995	0.976-1.015	0.648				
Sex (male *vs.* female)	-0.288	0.750	0.492-1.143	0.180				
Body mass index (kg/m^2)	0.042	1.042	0.994-1.093	0.085				
Comorbidity								
Diabetes mellitus	0.441	1.554	1.036-2.330	0.033				
Hypertension	0.035	1.035	0.653-1.641	0.883				
MELD score (point)	0.018	1.018	1.001-1.035	0.035				
Hepatic decompensation								
Encephalopathy (West-Haven criteria I or II)	0.417	1.517	0.828-2.778	0.177				
Varix	0.179	1.197	0.783-1.829	0.407				
Ascites	0.522	1.685	1.157-2.453	0.007				
Cardiac function								
Ejection fraction (%)	0.029	1.029	0.987-1.072	0.175				
Diastolic dysfunction	0.161	1.175	0.807-1.709	0.401				
Renal function								
eGFR (mL/min/1.73 m2)	0.002	1.002	0.997-1.006	0.510				
Laboratory variables								
Hemoglobin (g/dL)	-0.116	0.891	0.816-0.973	0.010				
WBC count (x 10^9/L)	0.017	1.017	0.986-1.049	0.288				
Albumin (g/dL)	-0.564	0.569	0.408-0.793	0.001				

Platelet count (x 10^9/L)	-0.007	0.993	0.989-0.997	0.001	-0.005	0.995	0.991-0.999	0.014
International normalized ratio	0.126	1.135	0.913-1.410	0.255				
Activated partial thrombin time (sec)	0.004	1.004	0.986-1.022	0.686				
D-dimer (mg/L) (dichotomized)								
Normal D-dimer level (0.5≤ mg/L)	Reference							
High D-dimer level (>0.5 mg/L)	1.408	4.392	1.984-9.722	<0.001	1.027	2.792	1.227-6.353	0.014
Sodium (mEq/L)	-0.020	0.980	0.947-1.014	0.240				
Potassium (mEq/L)	-0.138	0.871	0.636-1.192	0.388				
Calcium (mg/dL)	-0.116	0.891	0.697-1.138	0.355				
Glucose (mg/dL)	0.001	1.001	0.998-1.004	0.505				
Creatinine (mg/dL)	-0.135	0.873	0.723-1.055	0.161				
Ammonia (μg/dL)	0.001	1.001	0.999-1.003	0.338				
**Intraoperative recipient factor**								
Surgical duration (min)	0.001	1.001	0.999-1.003	0.361				
Postreperfusion syndrome	0.215	1.240`	0.852-1.805	0.261				
Average of vital signs								
MBP (mmHg)	-0.018	0.982	0.964-1.001	0.060				
HR (beats/min)	0.010	1.010	0.998-1.021	0.096				
CVP (mmHg)	0.043	1.044	0.985-1.107	0.148				
Mean lactate (mmol/L)	0.035	1.036	0.972-1.104	0.280				
Blood product transfusion (unit)								
Packed red blood cell	0.029	1.030	1.009-1.050	0.004				
Fresh frozen plasma	0.031	1.031	1.006-1.057	0.014				
Platelet concentrate	0.001	1.001	0.988-1.013	0.915				
Hourly fluid infusion (mL/kg/h)	0.011	1.011	0.994-1.029	0.198				
Hourly urine output (mL/kg/h)	-0.464	0.629	0.511-0.773	<0.001	-0.362	0.696	0.565-0.858	0.001

**Donor-graft factor**				
Age (years)	-0.003	0.997	0.980-1.014	0.740
Sex(male)	-0.100	0.905	0.585-1.400	0.653
GRWR (%)	0.226	1.253	0.857-1.832	0.244
Graft ischemic time (min)	0.003	1.003	1.001-1.004	<0.001
Fatty change (%)	0.006	1.006	0.979-1.006	0.669

Abbreviations: CI, confidence interval; CVP, central venous pressure; eGFR, estimated glomerular filtration rate; GRWR, graft-recipient weight ratio; HR, heart rate; MBP, mean blood pressure; MELD, Model for End-Stage Liver Disease; WBC, white blood cell.

Table S2. Comparison of the prevalences of AKI between the normal and high D-dimer groups of DM patients.

Group	Normal D-dimer (≤0.5 mg/L)	High D-dimer (>0.5 mg/L)	*p*
n	21	141	
AKI	3(14.3%)	45(31.9%)	0.099
**Group**	**Normal D-dimer (≤1.1 mg/L)**	**High D-dimer (>1.1 mg/L)**	***p***
**n**	**39**	**123**	
AKI	5(10.4%)	43(35%)	0.008

Abbreviations: AKI, acute kidney injury. NOTE: Values are expressed as numbers (with % proportion).

Table S3. Comparison of the D-dimer levels between the non-AKI and AKI groups of DM patients.

Group	non-AKI	AKI	*p*
n	114	48	
D-dimer (mg/L)	2.6(0.8-7.2)	4.2(2.4-8.8)	0.024

Abbreviations: AKI, acute kidney injury. NOTE: Values are expressed as medians (interquartile ranges).

Table S4. Comparison of the prevalences of AKI between the normal and high D-dimer groups of patients with hepatitis B virus.

Group	Normal D-dimer (≤0.5 mg/L)	High D-dimer (>0.5 mg/L)	*p*
n	72	277	
AKI	5(6.9%)	74(26.7%)	<0.001

Abbreviations: AKI, acute kidney injury. NOTE: Values are expressed as numbers (with % proportion).

Table S5. Comparison of the D-dimer levels between the non-AKI and AKI groups of patients with hepatitis B virus

Group	non-AKI	AKI	*p*
n	270	79	
D-dimer (mg/L)	1.85(0.5-4.9)	4.4(2.2-8.9)	<0.001

Abbreviations: AKI, acute kidney injury. NOTE: Values are expressed as medians (interquartile ranges).

Table S6. Comparison of the prevalences of AKI between the normal and high D-dimer groups of patients with heart disease (diastolic dysfunction).

Group	Normal D-dimer (≤0.5 mg/L)	High D-dimer (>0.5 mg/L)	*p*
n	36	222	
AKI	2(5.6%)	63(28.4%)	0.003

Abbreviations: AKI, acute kidney injury. NOTE: Values are expressed as numbers (with % proportion).

Table S7. Comparison of the D-dimer levels between the non-AKI and AKI groups of patients with heart disease (diastolic dysfunction).

Group	non-AKI	AKI	*p*
n	193	65	
D-dimer (mg/L)	2.5(0.7-6.0)	6.1(3.0-11.2)	<0.001

Abbreviations: AKI, acute kidney injury. NOTE: Values are expressed as medians (interquartile ranges).
